# Chronic fatigue syndrome 5 years after giardiasis: differential diagnoses, characteristics and natural course

**DOI:** 10.1186/1471-230X-13-28

**Published:** 2013-02-12

**Authors:** Kristine Mørch, Kurt Hanevik, Ann C Rivenes, Jørn E Bødtker, Halvor Næss, Bjarte Stubhaug, Knut-Arne Wensaas, Guri Rortveit, Geir E Eide, Trygve Hausken, Nina Langeland

**Affiliations:** 1 National Centre for Tropical Infectious Diseases, Department of Medicine, Haukeland University Hospital, Bergen, Norway; 2 Insitute of Medicine, University of Bergen, Bergen, Norway; 3 Division of Psychiatry, Haukeland University Hospital, Bergen, Norway; 4 Department of Neurology, Haukeland University Hospital, Bergen, Norway; 5 Department of Mental Health, Helse Fonna HF, Institute of Clinical Medicine, University of Bergen, Bergen, Norway; 6 Department of Global and Public Health, University of Bergen, Bergen, Norway; 7 Research Unit for General Practice, Uni Health, Bergen, Norway; 8 Centre for Clinical Research, Haukeland University Hospital, Bergen, Norway

**Keywords:** Giardia, Chronic fatigue syndrome, Depression, Anxiety, Sleep apnoea hypopnea syndrome

## Abstract

**Background:**

A high prevalence of chronic fatigue has previously been reported following giardiasis after a large waterborne outbreak in Bergen, Norway in 2004. The aim of this study was to describe and evaluate differential diagnoses and natural course of fatigue five years after giardiasis among patients who reported chronic fatigue three years after the infection.

**Methods:**

Patients who three years after *Giardia* infection met Chalder’s criteria for chronic fatigue (n=347) in a questionnaire study among all patients who had laboratory confirmed giardiasis during the Bergen outbreak (n=1252) were invited to participate in this study five years after the infection (n=253). Structured interviews and clinical examination were performed by specialists in psychiatry, neurology and internal medicine/infectious diseases. Fukuda et al’s 1994 criteria were used to diagnose chronic fatigue syndrome (CFS) and idiopathic chronic fatigue (ICF). Self-reported fatigue recorded with Chalder Fatigue Questionnaire three and five years after infection were compared.

**Results:**

53 patients were included. CFS was diagnosed in 41.5% (22/53) and ICF in 13.2% (7/53). Chronic fatigue caused by other aetiology was diagnosed in 24.5% (13/53); five of these patients had sleep apnoea/hypopnoea syndrome, six had depression and five anxiety disorder, and among these two had more than one diagnosis. Fatigue had resolved in 20.8% (11/53). Self-reported fatigue score in the cohort was significantly reduced at five years compared to three years (p<0.001).

**Conclusion:**

The study shows that *Giardia duodenalis* may induce CFS persisting as long as five years after the infection. Obstructive sleep apnoea/hypopnoea syndrome, depression and anxiety were important differential diagnoses, or possibly comorbidities, to post-infectious fatigue in this study. Improvement of chronic fatigue in the period from three to five years after giardiasis was found.

## Background

Chronic fatigue syndrome (CFS) is a disease with unknown pathogenesis that may be induced by infection, trauma, cancer or undefined conditions, and is characterized by abnormally long post-exertion rehabilitation, incomplete/unsatisfactory relief by rest or sleep, sleep disturbances, cognitive problems, tender lymph nodes, myalgia, headache, sore throat and other unspecific symptoms [1-3].

CFS has been reported to affect 10% following Epstein Barr virus (EBV) infection [4,5], and fatigue, depression and hair loss is a common syndrome following Dengue fever [6,7]. In controlled studies chronic fatigue has been reported after Lyme borreliosis [8], Parvovirus B19 infection [9], Q fever [10] and Ross River virus infection [4].

Graded exercise and cognitive behaviour therapy have been reported to be helpful treatment for some patients [11-13].

Diagnostic biological markers are not identified, and the diagnosis is based on the patient’s subjective report of a combination of characteristic symptoms, lasting for at least six months [1,3]. However, chronic fatigue severe enough to affect normal life is a common symptom in a range of chronic somatic and psychiatric disorders. Cancer; rheumatic, endocrine and neurologic disease; obstructive sleep apnoea/hypopnea syndrome (SAHS); depression and anxiety; obesity, drug side effects and alcohol or drug abuse are among disorders that have to be ruled out to establish the diagnosis CFS [1,3].

After a large outbreak of *Giardia duodenalis* in Bergen, Norway in 2004 [14], all laboratory confirmed cases have been followed up by repeated postal questionnaires [15,16], and 46% reported chronic fatigue three years after the *Giardia* infection [16].

In another cohort following the *Giardia* outbreak in Bergen, Næss et al. [17] has recently reported CFS among a selected group of patients referred to department of neurology by their doctor due to fatigue complaints one to three years after giardiasis; however, in that study differential diagnoses were not reported and clinical evaluation by internist and psychiatrist was not performed.

Unspecific chronic fatigue has previously been reported in 11% of the general Norwegian population [18], underlining that chronic fatigue after an infectious outbreak could possibly have many causes.

The main objective in the present study was to describe post-infectious fatigue, in the form of CFS and idiopathic chronic fatigue (ICF) as defined by Fukuda et al. [1], as well as differential diagnoses in the group of patients who had self-reported chronic fatigue three years after giardiasis. Secondary objectives were to describe the natural course of chronic fatigue based on self-reported fatigue scores [19] three and five years after the infection, and to identify factors associated with chronic fatigue in this cohort.

## Methods

### Study population

**Figure 1:**
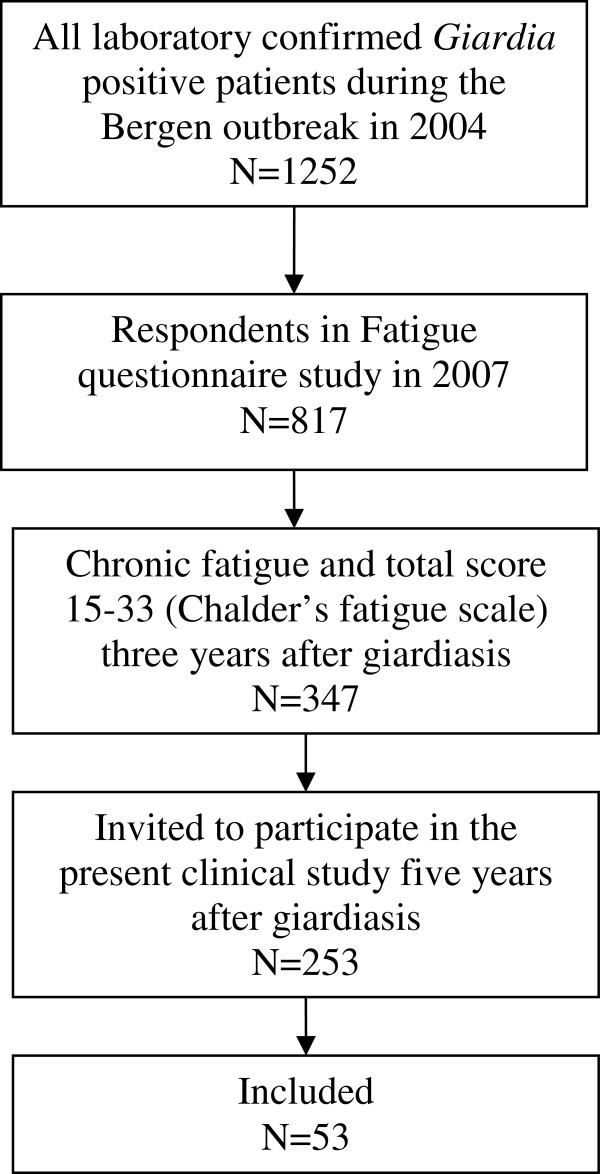
Flow chart showing inclusion of patients among laboratory confirmed cases of giardiasis during the outbreak in Bergen in 2004.

Subjects were included among patients who fulfilled the criteria for chronic fatigue in a postal questionnaire study three years after giardiasis among all laboratory confirmed *Giardia* cases in Bergen, Norway during an outbreak in 2004 [16]. A flow chart describing inclusion is shown in Figure 1. The questionnaire included Chalder Fatigue Questionnaire [19] consisting of 11 validated questions recording physical and cognitive manifestations of fatigue with four possible answers (1=“less than usual”, 2= “not more than usual”, 3=“more than usual” and 4=“much more than usual”) scored from 0 to 3, to give a possible total score between 0 and 33. Chronic fatigue is defined as at least six months duration of fatigue and a dichotomized total score ≥ 4 (answers 0 and 1 dichotomized into 0, and 2 and 3 into 1).

All patients who had chronic fatigue as well as a total score 15–33 (n = 347/817), were eligible to participate in the current study including clinical work-up and magnetic resonance imaging (MRI) of the brain. A cut-off of 15 was used in order to exclude those who had very mild self-reported fatigue between 11 and 14. As an optional part of the study, the patients were offered treatment consisting of randomisation into two different treatment programs performed by psychiatrists. Persons with age above 70 or less than 18 years and those who had moved away from the Bergen area were excluded for logistic reasons (n=88), six had died and the remaining 253 cases were invited to participate. Patients were included in the period of March 2009 to March 2010.

### Data collection and diagnostic evaluation

All participants were interviewed and clinically examined by specialists in neurology, psychiatry and internal medicine/infectious diseases. Structured interviews, which included Fukuda et al’s 1994 diagnostic criteria for CFS (CDC criteria) [1], as well as information regarding demographic data and premorbid and inter-current illness, were performed.

The Mini International Neuropsychiatric Interview-MINI PLUS, a short standardized diagnostic interview for DSM-IV and ICD-10 was used for evaluation of psychiatric diagnoses [20], and Montgomery and Åsberg depression rating scale (MADRS), which measure depression severity on a 10-item scale, was used for rating depressive symptoms [21].

A blood sample was taken from all patients, and screened for a panel of biochemical, microbiological and immunological parameters. Patients with positive *Borrelia* antibody test were asked to undergo a spinal tap with analysis for intrathecal *Borrelia* antibody production. Patients with elevated S-cortisol underwent a dexamethasone suppression test. Patients who reported snoring and sleep apnoea were referred to an ENT specialist for evaluation of SAHS. For other findings, patients were referred to relevant specialists according to clinical practice. Stool microscopy and 18 S PCR were performed to rule out chronic giardiasis.

Self - reported symptoms were recorded on Chalder Fatigue Questionnaire [19] and on Rome II irritable bowel syndrome (IBS) diagnostic questionnaires [22] under the supervision of a study nurse.

The diagnoses CFS or idiopathic chronic fatigue (ICF) were given according to the CDC criteria [1], where ICF defined a condition similar to CFS not fulfilling all criteria for a CFS case definition. Those who received other diagnoses explaining fatigue were categorised as “Chronic fatigue other aetiology”. When there was doubt if a disease represented comorbidity or an exclusion criterion for the diagnosis of CFS, it was categorised as “Chronic fatigue other aetiology”. Those who had experienced chronic fatigue following giardiasis earlier, but who no longer had fatigue affecting normal life, were categorised as “Recovered from fatigue”. CFS and ICF as defined by Fukuda et al. [1] were grouped in the analyses, and categorised as “*Giardia* induced CFS/ICF”. Diagnoses were evaluated based on all available data including structured interviews and clinical examinations. The psychiatric evaluation was performed by experienced psychiatrists among the authors. A second psychiatric evaluation among patients taking part in the treatment program was performed, with concordant results.

### Analyses

For each of the three response variables “*Giardia* induced CFS/ICF”, “Chronic fatigue other aetiology” and “Recovered from fatigue”, the possible risk factors were analysed as explanatory variables using the Chi square test for categorical variables and parametric and nonparametric one way (Kruskal-Wallis) anova test for continuous variables. Total fatigue scores three and five years after infection were compared using paired *t*-test, and the change from three to five years compared between the groups using the one way anova. P-values ≤ 0.05 were considered statistically significant. Statistical analyses were performed using SPSS 18.0.

### Ethics

The study was approved by the Regional Committee for Medical and Health Research Ethics and by the Ombudsman for Privacy in Research, Norwegian Social Science Data Services. Written informed consent was obtained from the participants.

## Results

Among those invited, 20.9% (53/253) chose to participate in the study. Mean age was 43.5 (median 41.4, range 20 – 69) years, and 77.4% (41/53) were women.

*Giardia* induced CFS/ICF was diagnosed in 54.7% (29/53) (Table 1). Chronic fatigue caused by other disease was diagnosed in 24.5% (13/53); five of these had SAHS, six had depression and five had anxiety disorder, and among these two had more than one diagnosis. Fatigue had resolved in 20.8% (11/53).

**Table 1:** **Distribution of diagnoses based on clinical evaluation five years after *Giardia duodenalis *infection in Bergen, Norway in 2004 in 53 cases reporting chronic fatigue three years after giardiasis**

	**Fatigue category**	**Diagnosis**
	**N (%)**	**N (%)**
**Total**	53 (100)	53 (100)
***Giardia *induced chronic fatigue**	29 (54.7)	
Chronic Fatigue Syndrome (CFS)^1^		22 (41.5)
Idiopathic Chronic Fatigue (ICF)^1^		7 (13.2)
**Chronic fatigue other aetiology**	13 (24.5)	
Obstructive sleep apnoea/hypopnoea syndrome (SAHS)		4 (7.5)
Depression		4 (7.5)
Anxiety disorder		3 (5.7)
Depression, anxiety and SAHS		1 (1.9)
Depression and anxiety disorder		1 (1.9)
**Recovered from fatigue**	11 (20.8)	

^1^Based on diagnostic criteria defined by Fukuda et al. (1994) [1].

**Figure 2:**
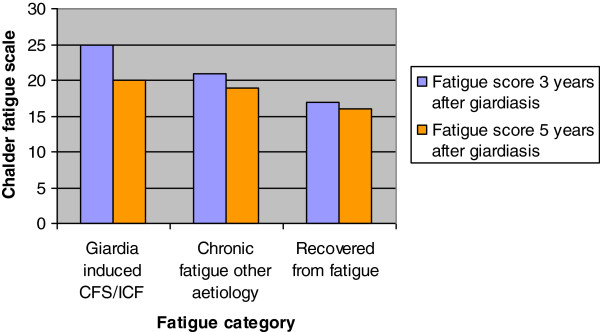
**Self reported Fatigue scores (mean) 3 and 5 years after giardiasis grouped by fatigue category, showing a significant reduction in fatigue score (p<0.001). **The scores at 3 and 5 years were compared using paired *t*-test.

Self-reported fatigue score in the whole cohort was significantly reduced at five years compared to three years (p < 0.001) (Figure 2). The score was reduced from 25 to 20 in the *Giardia* induced CFS/ICF category, from 21 to 19 among patients with other causes of chronic fatigue and from 17 to 16 among those who had recovered. However, these differences within the categories were not statistically significant.

Demographic characteristics and comorbidity associated with fatigue category is presented in Table 2. Age differed between the categories (anova p-value = 0.029), however in the pairwise comparison only the difference between those who had recovered from fatigue, being younger, and those who had chronic fatigue of other aetiology was significant (p = 0.027 in Sidak multiple comparison).

**Table 2:** Demographic characteristics and comorbidity among patients reporting chronic fatigue following giardiasis, associated with fatigue category five years after the infection

**Characteristics**	**All cases**	***Giardia *induced CFS/ICF**	**Chronic fatigue other aetiology**	**Recovered from fatigue**
	**N = 53**	**N = 29**	**N = 13**	**N = 11**
**Age,***mean (SD)*	43.5 (11.29)	44.4 (9.04)	47.9 (14.97)	35.9 (8.68)
**Gender**				
Female	41	23	12	6
Male	12	6	1	5
**On sick leave**^1^				
Yes	19	15	4	-
No	34	14	9	-
**Daily smoker**				
Yes	11	8	2	1
No	42	21	11	10
**BMI**^2^**,***mean (SD)*	25.6 (4.95)	24.5 (3.06)	28.34 (8.29)	25.5 (3.43)
**Alcohol consumption**^3^(u/m)				
*mean (SD)*	10.5 (10.18)	10.9 (11.04)	9.46 (9.40)	10.7 (9.68)
**Somatic comorbidity**				
Abdominal problems	45	23	13	9
IBS Rome II	31	17	7	6
Pregnancy	1	1	-	-
Asthma	11	4	5	2
Allergy	31	14	11	6
Diabetes type 2	2	-	2	-
Hypothyreosis	2	1	1	-
Myasthenia gravis	1	-	1	-
Epilepsy	1	1	-	-
Migraine	12	9	3	-
Psoriasis	3	1	2	-
Hypertension	3	1	2	-
Hypercholesterolemia	2	-	2	-
IgA nephritis	1	1	-	-
**Psychiatric comorbidity**				
Agarophobia	1	1	-	-
Dysthymia	3	2	1	-

Data are presented as number of cases (n), if not other stated. Each patient may have more than one diagnosis.

*Abbreviations*: CFS, Chronic Fatigue Syndrom; SD, standard deviation; BMI, body mass index; IgA, immunoglobuline A.

^1^Sick leave range; 20, 50, 60, 70 and 100%, grouped together.

^2^Missing values (excluded), 5.

^3^Missing values (excluded), 2.

Among patients with *Giardia* induced CFS/ICF, 51.7% were on sick leave, 30.8% were on sick leave among patients with chronic fatigue of other aetiology and none of the patients who had recovered from fatigue were on sick leave (p = 0.008).

Patients with SAHS had a mean BMI 29.80 (SD 9.01, range 23.7 – 43.1) compared to a mean of 25.18 (SD 4.40, range 18.5 – 40.7) among patients without SAHS; the difference was not statistically significant.

In the statistical analyses, no significant association was found between gender, smoking habit, alcohol consumption or comorbidity respectively and the fatigue categories.

Self-reported IBS according to Rome II criteria was recorded in 59.6% (31/52).

Asthma was recorded in 20.8% (11/53), allergy in 58.5% (31/53) and migraine in 22.6% (12/53). Asthma was significantly associated with elevated IgE level (data not shown). Comorbid agarophobia or dysthymia not severe enough to exclude the diagnosis of CFS were found in four patients.

Results from biochemical and microbiological blood tests were not significantly different in the three categories (data not shown). S-cortisol was elevated in five patients; none had findings suggesting Cushing’s disease, four had negative dexamethasone test and one positive dexamethasone test was explained by use of oral contraceptives. *Borrelia* IgG antibody was elevated in three patients without previous diagnosis of borreliosis, one had negative spinal fluid examination for intrathecal antibody production, two did not undergo spinal tap but had no findings suggesting neuroborreliosis on examination. Enterovirus antibody titers were elevated in 12 patients (range 16 – 64), and IgE was elevated in 11 patients. D-vitamin below normal range was found in 16 patients (range 17 – 48 nmol/L).

Previous morbidity before present investigation associated with fatigue categories is presented in Table 3. Four patients reported a self-limiting period of chronic fatigue prior to giardiasis, three of these following *Chlamydia psittachi, Yersinia enterocolitica* infection and mononucleosis respectively. Ten patients had previously had infections that are reported to have the potential of inducing chronic fatigue, namely borreliosis, mononucleosis or Dengue fever, without fatigue following these infections.

**Table 3:** Previous medical history among patients who reported chronic fatigue 3 years after giardiasis, associated with fatigue categories 5 years after the infection

**Previous history**	**All cases**	***Gxiardia *induced CFS/ICF**	**Chronic fatigue other aetiology**	**Recovered from fatigue**
	**N = 53**	**N = 29**	**N = 13**	**N = 11**
**Fatigue score at 3 years***mean, range (SD)*	23, 15–33 (5.80)	25, 15–33 (5.20)	21, 15–32 (6.02)	18, 15–22 (1.86)
**Fatigue before giardiasis, induced by**				
Mononucleosis	1	1	-	-
*Chlamydia psittachi*^*1*^	2	-	1	1
*Yersinia enterocolitica*^*1*^	1	-	1	-
Undefined	1	1	-	-
**Infections with reported potential to induce fatigue**				
Borreliosis	2	0	2	-
Mononucleosis	7	3	3	1
Dengue infection	1	1	-	-
**Previous diagnoses**				
Sleep apnoea^2^	1	1	-	-
Whip lash trauma	6	3	-	3
Musculoskeletal^3^	7	3	4	-
Thyreoiditis	1	1	-	-
Silicon breast implant	1	1	-	-
Meningitis	1	1	-	-
Pneumonia^4^	3	1	1	1
Pyelonephritis	1	1	-	-
Tonsillectomy	1	-	-	1
Cervical dysplasia	3	3	-	-
Syncope	2	1	1	-
Adipositas surgery	2	1	1	-
Severe abdominal diagn^5^	11	3	6	2
Infectious gastroenteritis	7	2	3	2
Irritable bowel syndrome	3	2	-	1
Cancer^6^	2	-	2	-
Depression	19	11	6	2
Anxiety disorder	2	-	1	1
Alcohol overuse	1	-	1	-

Data are presented as number of cases (n) if not other stated. Each patient may have more than one diagnosis.

^1^One patient had had fatigue after both *Yersinia* and *Chlamydia* infection.

^2^Recovered after adipositas surgery.

^3^Including the following diagnoses: Polyarthrosis, chronic single or multiple joint pain, fibromyalgia, cervical prolaps.

^4^Two patients had pneumonia due to *Chlamydia psittachi.*

^5^Including the following: Acute abdomen leading to hospitalisation; ulcus ventriculi or duodeni; inflammatory bowel disease; cholecysteoctomised; cholecystitis; cholecystolithiasis; appendicitis; faecal incontinence following haemorrhoid operation.

^6^Cancer ovarii; cancer mamma.

Fatigue score at three years differed between the categories (anova p-value <0.001), however in pairwise comparisons only the difference between *Giardia* induced CFS/ICF, having a higher score, and those who had recovered was significant (p<0.001 in Sidak multiple comparison).

A previous severe abdominal condition was significantly associated (p=0.031) with having chronic fatigue with other aetiology. In the statistical analyses, no other significant association was found between fatigue category and previous morbidity. Among all patients, 19 (35.8%) had previous depression and six (11.3%) had experienced whip lash trauma. Seven (13.2%) had a previous musculoskeletal disorder diagnosis (Table 3).

## Discussion

In this study, CFS/ICF was diagnosed in 54.7% among 53 patients investigated five years after *Giardia duodenalis* infection, who had reported chronic fatigue three years after the infection. As much as 25% of the patients in this cohort had other diagnoses possibly explaining chronic fatigue underlining the importance of evaluating differential diagnoses.

A significant reduction in self-reported fatigue at five years compared to three years after giardiasis was found, suggesting that chronic fatigue following giardiasis has a protracted but self-limiting course, also supported by the finding that 20.8% in the cohort who had experienced post-*Giardia* chronic fatigue earlier had recovered at five years.

All chronic fatigue patients not diagnosed as *Giardia* induced CFS/ICF had SAHS, depression or anxiety. According to Fukuda et al. (CDC criteria), sleep apnoea is an exclusion criteria for the diagnosis of CFS [1]. In a report from patients evaluated in a CFS referral clinic in UK, sleep apnoea was the most common somatic cause of fatigue wrongly diagnosed as CFS by general practitioners [23]. However, other researchers argue that SAHS is a common comorbidity rather than exclusion criterion in CFS. Polysomnographic evaluation revealed SAHS in 60% among patients with CFS in one report from Canada [24]. Further studies by their research group reported that treatment of SAHS with continuous positive airway pressure (CPAP) did not improve fatigue in patients with CFS and SAHS, supporting that SAHS was a comorbid disease and not an exclusion criterion to the CFS diagnosis [25]. Furthermore, both patients with CFS, and patients with SAHS without CFS, experienced worse sleep quality, fatigue and psychological functioning compared to a healthy control group [25], supporting our finding that SAHS is an important differential diagnosis to CFS but also may be a possible comorbidity.

Psychiatric illness in chronic fatigue may be a comorbid condition or a consequence due to reduced quality of life, rejection and stigmatization often experienced by these patients. Severe psychiatric illness is defined as an important exclusion criterion to the diagnosis of CFS [1], as such disorders will often have chronic fatigue as implicit symptom of the illness. However, depression and anxiety are commonly misdiagnosed as CFS [23,26] with potentially severe consequences due to lack of adequate diagnosis and treatment. It may be difficult to differentiate psychiatric illness from secondary psychological symptoms found in CFS [25], and evaluation by a psychiatrist may be needed in order to establish the correct diagnosis and treatment.

In our study, CFS was excluded when SAHS, depression and anxiety were diagnosed, although it should be taken into account that giardiasis may have contributed to or triggered chronic fatigue also among these patients.

A high prevalence of IBS [16,27] and a highly significant association between chronic fatigue and IBS has previously been reported among patients who had laboratory confirmed giardiasis during the Bergen outbreak [16]. A high level of IBS comorbidity was confirmed in the present study (Table 2). Interestingly, a high prevalence of comorbid allergy (58.5%) and asthma (20.3%) was recorded in the present study, supporting the possible association between chronic fatigue and atopic disease reported and discussed previously [28].

Those who had recovered from chronic fatigue were significantly younger than those who had chronic fatigue with other aetiology, supporting that higher age could be a risk factor for chronic fatigue as previously reported after the *Giardia* outbreak [15]. No potential risk factors were found to be significantly associated with CFS/ICF compared to the other categories. However, the limited number of patients should be taken into account when interpreting the analyses of factors potentially associated with CFS/ICF in this study.

The participation rate was relatively low (21%), possibly influenced by factors such as lack of energy to participate in the extensive work up, interpretation of invitation to psychiatric treatment as an unwanted insinuation of chronic fatigue having psychiatric aetiology, recovery from chronic fatigue or severe fatigue hampering the patient’s ability to participate. However, the lack of representativeness among all *Giardia* positive individuals during the Bergen outbreak does not have important implications for the interpretation of the results in this clinical study, since it describes CFS and differential diagnoses in a cohort of self-reported fatigue following giardiasis, and does not aim at identifying the prevalence of post-*Giardia* CFS in the general population.

A strength in this study is the thorough clinical evaluation as basis for the CFS/ICF diagnosis. In a study by Næss et al. [17] reporting CFS in 60% one to three years after giardiasis during the Bergen outbreak, patients were selected among those referred to department of neurology and not by invitation among all laboratory confirmed cases reporting chronic fatigue as in the present study and results are therefore not comparable. Næss et al. report a high level of depression and anxiety among their patients, however, evaluation by psychiatrist was not performed to evaluate these conditions as differential diagnoses to CFS.

The pathophysiology in post-infectious chronic fatigue is not well described, and in *Giardia* induced chronic fatigue even less, but symptom pattern as well as infectious aetiology would support the hypothesis that immunological dysfunction could play a role. In a recent report from the present cohort investigating previously reported markers of immune dysfunction in CFS, a decreased level of natural killer cells was found among CFS patients, suggesting a possible immunological abnormality that should be investigated further [29].

## Conclusions

This study confirms that *Giardia duodenalis,* a parasite previously regarded to be responsible for mainly uncomplicated infections, is capable of inducing chronic fatigue syndrome that may persist as long as five years after the infection. Improvement of fatigue symptoms at five years compared to three years after giardiasis was found. Obstructive sleep apnoea/hypopnoea syndrome, depression and anxiety disorders were important differential diagnoses, or possible comorbidities, to post-giardiasis CFS/ICF in this cohort.

## Competing interests

The authors declare that they have no competing interests.

## Authors’ contributions

Clinical evaluation of patients was performed by KM, KH, ACR, BS, JEB and HN. Data on self-reported fatigue scores and IBS criteria were collected by KH and KAW. GEE and KM did the statistical analyses. KM wrote the first draft of the manuscript. All authors contributed to planning of the study, interpretation of results and revision of the manuscript and approved the final version.

## Pre-publication history

The pre-publication history for this paper can be accessed here:

http://www.biomedcentral.com/1471-230X/13/28/prepub
